# Extreme resistance to weak-acid preservatives in the spoilage yeast *Zygosaccharomyces bailii*^☆^

**DOI:** 10.1016/j.ijfoodmicro.2013.06.025

**Published:** 2013-08-16

**Authors:** Malcolm Stratford, Hazel Steels, Gerhard Nebe-von-Caron, Michaela Novodvorska, Kimran Hayer, David B. Archer

**Affiliations:** a School of Biology, University of Nottingham, University Park, Nottingham NG7 2RD, United Kingdom; b Mologic Ltd., Bedford Technology Park, Thurleigh, Bedford MK44 2YP, United Kingdom

**Keywords:** Spoilage, Sorbic acid, Acetic acid, Intracellular pH, Population heterogeneity, Cross resistance

## Abstract

Weak-acid preservatives, such as sorbic acid and acetic acid, are used in many low pH foods to prevent spoilage by fungi. The spoilage yeast *Zygosaccharomyces bailii* is notorious for its extreme resistance to preservatives and ability to grow in excess of legally-permitted concentrations of preservatives. Extreme resistance was confirmed in 38 strains of *Z. bailii* to several weak-acid preservatives. Using the brewing yeast *Saccharomyces cerevisiae* as a control, tests showed that *Z. bailii* was ~ 3-fold more resistant to a variety of weak-acids but was not more resistant to alcohols, aldehydes, esters, ethers, ketones, or hydrophilic chelating acids. The weak acids were chemically very diverse in structure, making it improbable that the universal resistance was caused by degradation or metabolism. Examination of *Z. bailii* cell populations showed that extreme resistance to sorbic acid, benzoic acid and acetic acid was limited to a few cells within the population, numbers decreasing with concentration of weak acid to < 1 in 1000. Re-inoculation of resistant sub-populations into weak-acid-containing media showed that all cells now possessed extreme resistance. Resistant sub-populations grown in any weak-acid preservative also showed ~ 100% cross-resistance to other weak-acid preservatives. Tests using ^14^C-acetic acid showed that weak-acid accumulation was much lower in the resistant sub-populations. Acid accumulation is caused by acid dissociation in the higher pH of the cytoplasm. Tests on intracellular pH (pH_i_) in the resistant sub-population showed that the pH was much lower, ~ pH 5.6, than in the sensitive bulk population. The hypothesis is proposed that extreme resistance to weak-acid preservatives in *Z. bailii* is due to population heterogeneity, with a small proportion of cells having a lower intracellular pH. This reduces the level of accumulation of any weak acid in the cytoplasm, thus conferring resistance to all weak acids, but not to other inhibitors.

## Highlights

• *Z. bailii* resistance to weak acids was due to population heterogeneity.
• Resistant *Z. bailii* sub-populations were cross resistant to all other weak acids.
• Weak acid accumulation is determined by cell internal pH.
• Resistant *Z. bailii* sub-populations had a lower internal pH, ~ 0.6 pH units.
• Resistant *Z. bailii* sub-populations absorbed 3-fold less ^14^C acetic acid.

## 1 Introduction

Many foods form ideal substrates for the growth of fungi, both yeasts and moulds, due to their carbohydrate, protein and vitamin content. If left untreated, fungal growth will result in spoilage, due to alterations in visual appearance, texture, taste, aroma, and the formation of fungal biomass and in some cases, a variety of mycotoxins. In order to prevent microbial spoilage, many foods are sterilised using heat, while others are treated with preservatives of proven safety of which the great majority are weak-acids. Soft drinks may contain limited concentrations of sorbic acid (2,4-hexadienoic acid) or benzoic acid (Anon., 1995) while acetic acid, commonly used as a preservative in salad dressings, pickles and vinegars, is legally recognised as an acidulant within the EU (Anon., 1995).

Preservatives inhibit the great majority of yeast and mould species, but a few species are able to proliferate in preserved foods (Pitt and Hocking, 1997). These are the spoilage fungi, and their physiological properties largely define their spoilage behaviour. The most dangerous spoilage yeasts (Group 1) were characteristically preservative-resistant (Davenport, 1996), osmotolerant, vitamin-requiring and highly fermentative, leading to excessive gas formation, bottle explosions, and occasional physical injury (Grinbaum et al., 1994). The majority of yeast species were Group 3 (hygiene indicators, not causing spoilage) while Group 2 were opportunistic yeasts able to cause spoilage following mistakes in manufacturing (Davenport, 1997, 1998). The most notorious of the Group 1 spoilage fungi, due to its outstanding degree of preservative resistance, was a yeast species known as *Zygosaccharomyces bailii*.

*Z. bailii*, reviewed by Thomas and Davenport (1985) and James and Stratford (2011), is a yeast naturally-occurring in mummified dried fruits, readily forming moderately heat-resistant ascospores. It is osmotolerant (Tilbury, 1976) and grows preferentially on fructose (Emmerich and Radler, 1983). This species is similar in some respects to the brewing yeast *Saccharomyces cerevisiae*, fermenting in aerobic conditions (Merico et al., 2003; Rodrigues et al., 2001) and in anaerobic conditions with suitable nutritional supplementation (Rodrigues et al., 2001). Spoilage by *Z. bailii*, reviewed by Fleet (1992), includes soft drinks (Sand, 1973), cordials and tomato sauce (Pitt and Richardson, 1973), high-sugar syrups (Tokuoka, 1993), acetic preserves (Dennis and Buhagiar, 1980), wine (Goswell, 1986) and cider (Beech, 1993). *Z. bailii* is reported to be highly resistant to sorbic, benzoic, acetic and propionic acids (Ingram, 1960; Malfeito-Ferreira et al., 1997; Neves et al., 1994; Pitt, 1974) and to sulphite (Goswell, 1986; Goto, 1980; Hammond and Carr, 1976) and hydroxycinnamic acids (Stead, 1995). It is also reported to be resistant to ethanol and other alkanols (Fujita et al., 2008; Goswell, 1986; Thomas and Davenport, 1985) and to carbonation (Ison and Gutteridge, 1987) and low pH (Betts et al., 1999).

The causes of resistance in *Z. bailii* have been investigated on several occasions and the overall results can be circumscribed by two possible hypotheses; 1. degradation and metabolism of the preservatives, and 2. efflux pumps removing preservatives. Metabolism of acetic acid by *Z. bailii* in the presence of glucose has been demonstrated (Guerriero et al., 2012; Rodrigues et al., 2012; Sousa et al., 1996, 1998) as have degradation of benzoic acid and sorbic acid (Ingram, 1960; Mollapour and Piper, 2001). However, removal of sufficient acids to affect resistance has not been confirmed and earlier studies (Warth, 1977) concluded that weak-acid metabolism was insufficient to explain resistance in *Z. bailii*. Efficient decarboxylation of weak-acid preservatives using the fungal Pad decarboxylation system was shown not to occur in *Z. bailii* (Stratford et al., 2007). Efflux of preservatives due to a “sorbate pump” was proposed by Warth (1977, 1988). It has been shown that lipophilic weak acids enter the cell rapidly by simple diffusion (Stratford and Rose, 1986; Warth, 1989a) but are concentrated because of the higher pH of the cytoplasm causing acid dissociation into their respective anions. This concentration effect led to early claims that uptake was an active transport process (Macris, 1975). At higher pH, there is evidence of mediated uptake of low concentrations of acetate (Sousa et al., 1996). Pre-growth of *Z. bailii* cells in benzoic or propionic acids, however, resulted in a 40% slower uptake of preservatives, which was proposed to be the result of active acid efflux from adapted cells (Warth, 1977, 1989a). Preservative resistance in 23 other yeast species was also correlated with uptake rate of propionic acid (Warth, 1989b). A similar sorbate efflux system has been reported in *S. cerevisiae*, encoded by the *PDR12* gene (Piper et al., 1998). However, it has been shown that such a system is not induced in *Z. bailii* in response to preservatives (Piper et al., 2001). Therefore, the causes of extreme preservative resistance in *Z. bailii* remain unresolved.

In this paper, we set out to investigate the causes of weak-acid preservative resistance in *Z. bailii*. Population heterogeneity to weak acids was also examined in light of an earlier study showing that only a very small proportion of the population of *Z. bailii* cells were resistant to sorbic acid (Steels et al., 2000).

## 2 Materials and methods

### 2.1 Strain variation in *Z. bailii*

The yeast strains used in this study are listed in Table 1 together with their source of isolation. The identity of all strains was confirmed by sequencing the D1/D2 region of the 26S rDNA using the methods described by Kurtzman (2003). Yeast strains were stored in glycerol on ceramic beads at –80 °C (Microbank™), and maintained short term on MEA (malt extract agar, Oxoid) slopes at 4 °C.

The growth medium used to assess strain variation was YEPD, glucose 20 g/l, bacteriological peptone (Oxoid) 20 g/l, and yeast extract (Oxoid) 10 g/l, adjusted to pH 4.0 with 10 M HCl prior to heat sterilisation. Starter cultures comprised 10 ml YEPD pH 4.0 in 28 ml McCartney bottles, inoculated and incubated for 48 h at 25 °C.

Resistance to weak-acid preservatives was determined by the minimum inhibitory concentration (MIC) of each acid to completely inhibit growth. Series of McCartney bottles were prepared with 10 ml aliquots of YEPD, each containing a progressively higher concentration of preservative. The pH of all media was back-titrated to pH 4.0 following acid addition. Bottles were inoculated with yeast at a final concentration of 10^3^ cells/ml and incubated for 14 days at 25 °C. The MIC was the lowest concentration of preservative at which no growth was detectable at 14 days.

### 2.2 Resistance of *Z. bailii* to weak acids and inhibitors

Tests were carried out using *Z. bailii* (NCYC 1766) and *S. cerevisiae* (BY4741) on resistance to 87 chemical inhibitors, including lipophilic and hydrophilic weak-acids, alcohols, chelating acids, ethers, aldehydes and esters (Supplementary Data Table 1). For the majority of inhibitors, 500 mM stock solutions were prepared in methanol, and added to YEPD aliquots at increasing concentrations. Bottles were inoculated at 10^3^ cells/ml and incubated for 14 days at 25 °C. The MIC was the lowest concentration of preservative at which no growth was detectable at 14 days.

### 2.3 Population heterogeneity in resistance of *Z. bailii* and *S. cerevisiae*

In preserved soft drinks, spoilage yeasts fall to the bottom of bottles. To imitate these conditions in the laboratory, resistance of individual cells was determined by colony growth in static liquid culture. Yeast cells in liquid media subside to the bottom at ~ 5 mm/h and in static conditions form discrete, countable yeast colonies under the liquid, at the bottom of Petri dishes or bottles. However, any later disturbance will cause yeasts to disperse, merging the colonies. To prevent agitation and maintain separate colonies, yeasts were grown in liquid culture in flat-bottomed 96-well microtitre plates (Steels et al., 2000).

Starter cultures of *Z. bailii* (NCYC 1766) and *S. cerevisiae* strain (BY4741) in YEPD pH 4.0 were accurately counted by haemocytometer and serially diluted in YEPD to 10^4^ cells/ml. 20 ml aliquots of YEPD containing progressively higher concentration of sorbic acid (0–7 mM) were inoculated at 15–30 cells/ml final concentration and dispensed into microtitre plates at 200 μl/well (maximum 3–6 colonies/well). Plates were sealed, lidded, double-bagged to prevent evaporation, and incubated at 25 °C for 14 days. Yeast colonies/well were recorded every 2 days, as high levels of preservatives progressively slow yeast growth. For each sorbic acid concentration, five replicate microtitre plates were inoculated, and the procedure repeated using four separate yeast starters. After 14 days, the total colony number at each sorbic acid concentration was recorded, and expressed in proportion to the colony number growing in YEPD pH 4.0 without sorbic acid.

### 2.4 Measuring resistance of sub-populations of *Z. bailii*

Tests of population heterogeneity, Section 2.3, showed very few *Z. bailii* (NCYC 1766) colonies growing at high concentrations of sorbic acid. Single colonies growing in 6 mM sorbic acid after 14 days, were mixed in their microtitre plate wells, and counted accurately by haemocytometer. Cultures were then serially diluted to 10^4^ cells/ml in YEPD pH 4.0 containing 6 mM sorbic acid. 20 ml aliquots of YEPD containing progressively higher concentration of sorbic acid (0–8 mM) were inoculated at 15–30 cells/ml and dispensed into microtitre plates at 200 μl/well (maximum 3–6 colonies/well). Plates were sealed, lidded, double-bagged to prevent evaporation, and incubated at 25 °C for 14 days.

These experiments were repeated using benzoic acid over the range 0–8 mM and acetic acid, 0–450 mM, using *Z. bailii* NCYC 1766 in YEPD corrected to pH 4.0. Resistant of sub-populations were selected of single colonies growing in 8 mM benzoic acid or in 350 mM acetic acid at 14 days, and re-inoculated.

### 2.5 Growth rates of *Z. bailii* in increasing concentrations of preservatives

The rates of growth *Z. bailii* (NCYC 1766) in increasing concentrations of weak-acid preservatives was monitored in the 96-well microtitre plates by the time required for the yeast colonies to reach 0.5–1 mm in size. In the absence of preservatives, this required only 2–3 days incubation. At higher concentrations of preservatives, the incubation time required increased up to 12–14 days.

### 2.6 Cross-resistance in sub-populations of *Z. bailii*

As previously described in Section 2.4, single colonies of *Z. bailii* (NCYC 1766) growing in 6 mM sorbic acid, 8 mM benzoic acid or 350 mM acetic acid after 14 days, were mixed in the microtitre well and accurately counted by haemocytometer. Each was then serially diluted in YEPD containing the same weak acid concentration to 10^4^ cells/ml. Each was then cross-inoculated into all combinations of weak acids, at 15–30 cells/ml into 20 ml aliquots of YEPD containing sorbic acid (0–8 mM), benzoic acid 0–8 mM and acetic acid 0–450 mM. These were then dispensed into microtitre plates at 200 μl/well (maximum 3–6 colonies/well). Plates were sealed, lidded, double-bagged to prevent evaporation, and incubated at 25 °C for 14 days.

### 2.7 Measurement of cellular internal pH by flow cytometry

The method used for determination of cellular internal pH by flow cytometry was a modification of the method described in Stratford et al. (2009). Exponentially-growing yeast cells were obtained from shake flasks at OD 1.65–2.2 (measured OD 0.15–0.2 following an 11-fold dilution in water). *Z. bailii* (NCYC 1766) and *S. cerevisiae* (BY4741) were cultured in 40 mls YEPD pH 4.0 in 100 ml conical flasks shaken for 12–16 h at 130 rpm and 25 °C. Sub-populations in 6 mM sorbic acid, 8 mM benzoic acid and 350 mM acetic acid in microtitre plates were inoculated into 40mls of the same media and shaken for 5 days (OD 0.15 × 11). Control samples were tested in microtitre plates at 0, 6 mM sorbic acid, 8 mM benzoic acid or 350 mM acetic acid to confirm that these populations were ~ 100% resistant to preservatives. CFDASE (carboxyfluoresceindiacetate succimidyl ester) was added to yeast in the growth media at 10 μg/ml final concentration and cells were incubated at 25 °C for 30 min for uptake of the CFDASE. Uncharged CFDASE, colourless and non-fluorescent, passes into the cell where it is cleaved intracellularly by esterases. The fluorescent succimidyl ester binds to proteins, ensuring retention of the dye within the cell. The internal pH of populations of individual fluorescent cells was determined from the linear ratio of the 575 nm (largely pH-independent) and 525 nm (pH-dependent) emission signals. Calibration was carried out using cells of defined intracellular pH, permeated using 2 mM 2, 4-dinitrophenol in 0.7 M acetate/100 mM succinate/100 mM KH_2_PO_4_ buffer, and 100 μM nigericin in the same permeating buffer.

### 2.8 Measurement of acetic acid uptake

Weak acid transport was tested using a modification of the method described by Stratford and Rose (1986). Exponentially-growing yeast cells, *Z. bailii* (NCYC 1766), were obtained from 40 ml shaken cultures, YEPD pH 4.0, at OD 1.65–2.2. Sub-populations were grown in 6 mM sorbic acid for five days as described in Section 2.7. Yeast concentrations were determined by optical density and converted to dry weight using calibration curves. The uptake medium consisted of 6 ml yeast growth culture in YEPD equilibrated at 25 °C for 3 min. Uptake was initiated by addition of acetic acid (30 mM final concentration) and 5 μCi ^14^C-acetic acid (PerkinElmer, UK). Samples, 1 ml, were removed over 1–10 min, and were rapidly filtered through 28 mm cellulose nitrate filters, pore size 0.45 μm. Filters were pre-washed with 3 ml YEPD containing 30 mM acetic acid pH 4.0 (no ^14^C). Immediately after sample filtration, filters were again rapidly washed with 3 mls YEPD containing 30 mM acetic acid, pH 4.0. Filters were placed into 5 ml ScintiSafe 3 liquid scintillation cocktail (Fisher Scientific, UK) and samples were counted using a Packard TRI-CARB 2100 TR liquid scintillation analyser.

## 3 Results

### 3.1 Strain variation in *Z. bailii*

A total of 38 strains of *Z. bailii* were initially tested, firstly to confirm preservative resistance, secondly to select typical strains, and thirdly to examine variations in preservative resistance between strains. Strains were selected from a global distribution, predominantly from a variety of spoiled foods and beverages (Table 1) but also included factory isolates and strains from fermented Kombucha tea, which frequently contains high levels of acetic acid. The identity of all strains was confirmed as *Z. bailii* by D1/D2 rDNA sequencing (Kurtzman, 2003). Two strains of *S. cerevisiae* were also included as reference strains. Previous research had shown these strains to be typical representatives of *S. cerevisiae* with respect to weak-acids (Stratford et al., 2013). Tests were carried out on the resistance of strains to sorbic, benzoic and acetic acids in YEPD at pH 4.0 (Table 1). Results showed variation in the resistance of *Z. bailii* strains to sorbic acid, MIC from 4.5 mM to 9.5 mM, MIC of benzoic acid 6.3 mM to 11 mM and the MIC of acetic acid, from 275 mM to 580 mM. In all strains examined, sorbic acid inhibited growth at a much lower concentration than acetic acid. The mean *Z. bailii* MIC of sorbic acid was 7.1 mM at pH 4.0, benzoic acid MIC 8.75 mM and mean acetic acid MIC was 466 mM. The resistance of *S. cerevisiae* strains to preservatives was far lower, with MICs in the region of 3 mM for sorbic acid or benzoic acid and 130 mM for acetic acid. The origin of yeast strains appeared unrelated to their preservative-resistance characteristics. Overall, this confirms that all strains of *Z. bailii* tested showed extreme resistance to sorbic, benzoic and acetic acid, and enabled selection of typical representative strains.

### 3.2 Resistance of *Z. bailii* to other weak acids and inhibitors

Tests were carried out using a single strain of *Z. bailii* (NCYC 1766) and a control *S. cerevisiae* strain (BY4741), on resistance to a wide variety of chemical inhibitors, 87 in total, including lipophilic and hydrophilic weak-acids, alcohols, chelating acids, ethers, aldehydes and esters. This was intended to map the physical and chemical characteristics of compounds to which *Z. bailii* is resistant. MIC tests were carried out for all compounds and the ratio of MICs between *Z. bailii* and *S. cerevisiae* was used as an indicator of extreme resistance. The data are summarised in Table 2 and the full data are recorded in Supplementary Data Table 1. Overall, *Z. bailii* was consistently far more resistant than *S. cerevisiae* to weak acids (30 tested), with a mean ratio of 2.98, indicating that the molar inhibitory concentrations of weak acids were 3-fold higher for *Z. bailii* than *S. cerevisiae*. These weak acids are very diverse in structure and properties, ranging from 2,4-dinitrophenol to 3-phenylpropiolic acid and adamantanecarboxylic acid. However, *Z. bailii* did not show any consistent increase in resistance over *S. cerevisiae* to aldehydes, alcohols, ketones, ethers or esters. Neither did *Z. bailii* show resistance to non-permeating (Conway and Downey, 1950) chelating acids, such as citric acid, succinic acid or EDTA (Stratford, 1999), which inhibit by absorbing minerals from the growth media.

**Fig. 1:**
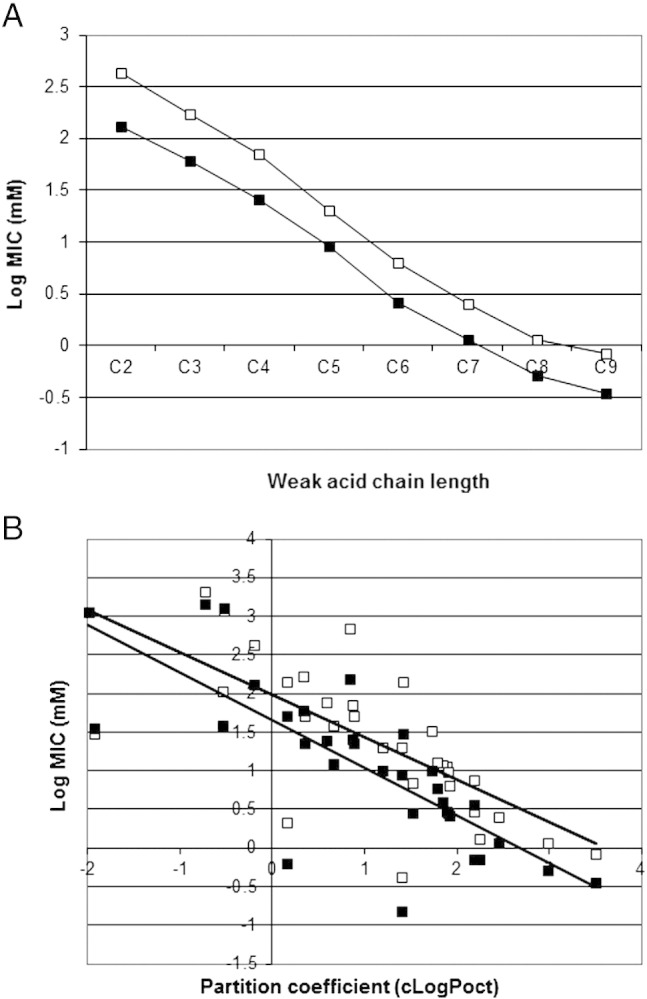
A. Resistances (MIC) to the aliphatic acid series, C2 acetic acid–C9 nonanoic acid by *S. cerevisiae* BY4741 (closed squares) and *Z. bailii* NCYC 1766 (open squares). Data, are presented as a log (base 10) scale plot of mean MIC values, measured in triplicate in YEPD pH 4.0 over 14 days at 25 °C. B. Scatter plot of resistance (MIC) to a variety of weak acids (Supplementary Data Table 1) in *S. cerevisiae* BY4741 (closed squares) and *Z. bailii* NCYC 1766 (open squares) plotted against the partition coefficient (cLogP_oct_). Data presented are log (base 10) scale plots of MIC values, measured in duplicate in YEPD pH 4.0 over 14 days at 25 °C. Linear regressions are *Z. bailii* R^2^ 0.707 and *S. cerevisiae* R^2^ 0.763.

The chemical properties of the weak-acids to which *Z. bailii* showed extreme resistance were examined further. The aliphatic acid series from acetic acid to nonanoic acid all showed greater resistance by *Z. bailii* than *S. cerevisiae* but the overall pattern of resistance did not change with increasing lipophilicity (Fig. 1A). There was an obvious, near logarithmic, fall in the MIC value with increasing chain length for both yeast species, which closely corresponds with the lipid solubility (partition coefficient—clogP_oct_). However, the ratio of resistance between the yeast species did not change with chain length. Examination of resistance to all other weak-acids in *Z. bailii* and *S. cerevisiae*, showed similar results. The data are presented as a scatter-plot in Fig. 1B. Despite the variations in pK_a_ values between the different acids, the overall trend was that more hydrophobic acids with a higher partition coefficient were more toxic, with a lower MIC. However, the linear regression plots of *Z. bailii* and *S. cerevisiae* were almost parallel, showing no relative increase in *Z. bailii* resistance with hydrophobicity, as would be expected if resistance was due to alteration in membrane composition. Similarly, resistance due to altered membrane composition would also be expected to affect hydrophobic alcohols, ketones, esters and ethers in addition to weak acids. The data in Table 2 clearly show this not to be the case.

### 3.3 Population heterogeneity in resistance of *Z. bailii* and *S. cerevisiae*

**Fig. 2:**
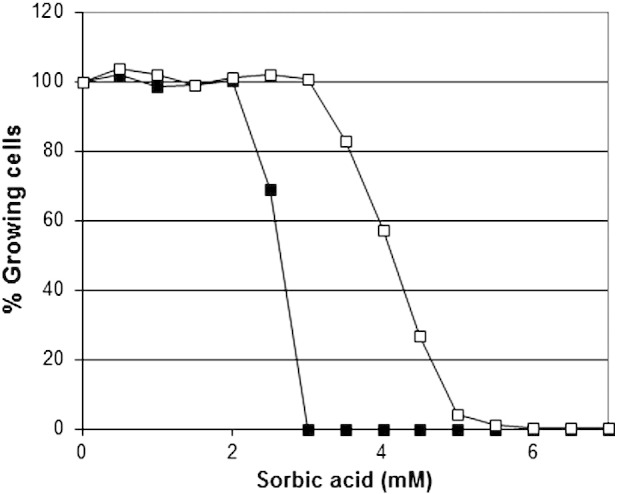
Proportion of growing cells within populations of *S. cerevisiae* BY4741 (closed squares) and *Z. bailii* NCYC 1766 (open squares) exposed to sorbic-acid, measured in YEPD pH 4.0 over 14 days at 25 °C. Populations of > 8000 cells were grown in 96-well microtitre plates, at 300–600 cells/plate. The slope of the “tail” when the proportion of growing cells is reduced from 100% to zero provides a measure of heterogeneity within the population (Avery, 2006).

It has been previously shown that resistance to sorbic acid in the spoilage yeast *Z. bailii* was largely due to small numbers of highly resistant cells within the cell population (Steels et al., 2000). Tests were therefore carried out to examine the impact of population variability to the sensitivities to sorbic acid, benzoic acid and acetic acid in *Z. bailii* strain NCYC 1766 (Fig. 2) using cell viability in liquid media. Results from populations of > 1000 cells showed that all *Z. bailii* cells were able to grow in sorbic acid over the range of 0–3 mM. However, a declining proportion of cells were able to grow at concentrations up to 7 mM, forming a long “tail” of sorbic-acid-resistant cells. Only ~ 1 cell in 8000 was able to grow in 7 mM sorbic acid. This is in close-agreement with the sorbic acid MIC of 7.62 mM for inocula of 10^4^ cells of strain NCYC 1766 (Table 1). In contrast, the *S. cerevisiae* cell population was 100% resistant up to 2 mM sorbic acid but with only a short “tail” of resistance up to 3 mM. Similar results were obtained for both benzoic acid and acetic acid, showing that extreme acid resistance in *Z. bailii* was most probably due to a small proportion of the population. It was noted that the resistant “tail” in acetic acid was substantially longer, than that formed in sorbic acid or benzoic acid. The existence of a resistant sub-population may explain why tests on whole *Z. bailii* populations would fail to reveal the causes of resistance in *Z. bailii*.

### 3.4 Resistant sub-populations of *Z. bailii*

**Fig. 3:**
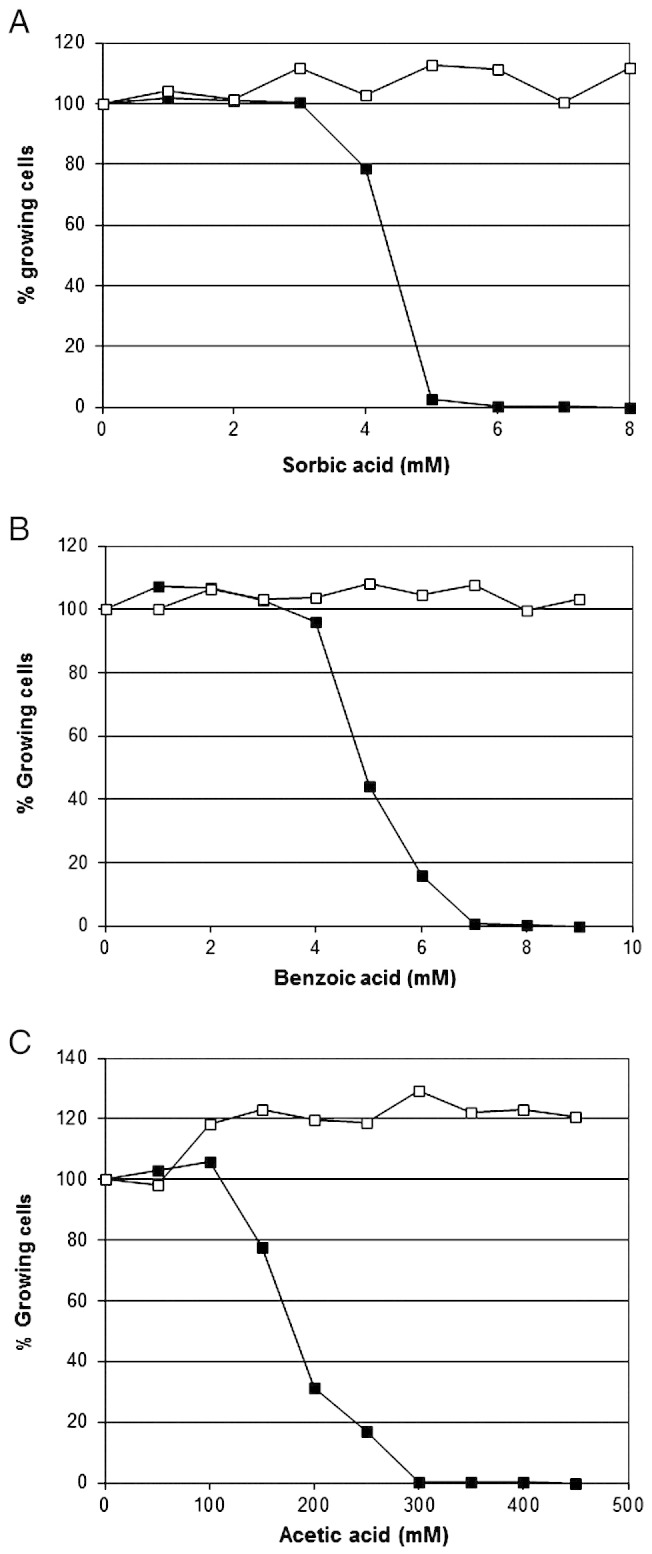
A. Comparison of resistance of sub-populations (6 mM sorbic acid) to normal populations of *Z. bailii*. Resistance of individual cells in normal populations of *Z. bailii* NCYC 1766 (closed squares) and individual cells in sub-populations pre-grown for 14 days in 6 mM sorbic acid (open squares), was measured in YEPD pH 4.0 over 14 days at 25 °C. Populations of > 8000 cells were grown in 96-well microtitre plates, at 300–600 cells/plate. B. Comparison of resistance of sub-populations (8 mM benzoic acid) to normal populations of *Z. bailii*. C. Comparison of resistance of sub-populations (350 mM acetic acid) to normal populations of *Z. bailii*.

Cell suspensions were prepared of the sub-populations of *Z. bailii* from the 6 mM sorbic acid microtitre plates. These were directly re-inoculated, without washing or sorbic acid removal, into media containing increasing levels of sorbic acid, and the percentage of the population able to grow was again determined at each level of preservative. It was found that near 100% of the cell population was now able to grow in sorbic acid up to 8 mM (Fig. 3A). These experiments were repeated using cells cultured from *Z. bailii* sub-populations growing in 8 mM benzoic acid and from 350 mM acetic acid. Again, near 100% of the cell populations were now able to grow in 9 mM benzoic acid or 450 mM acetic acid respectively (Fig. 3B; C). It was noted that sub-populations from 350 mM acetic acid showed 100% viability in high levels of acetic acid, but that a proportion, ~ 20%, failed to grow when inoculated into media lacking acetic acid. Since the proportion of cells that grew was expressed as a percentage of the cell population in the absence of sorbic acid, this caused an apparent 120% cell viability at higher acetic acid concentrations. We speculate that this loss of viability was due to cytoplasmic alkalinisation caused by the large acetic acid efflux. Extreme resistance in the sub-populations was shown not to be genetically heritable, since if these sub-populations were grown overnight in YEPD pH 4.0 containing no preservatives and were then re-inoculated into media containing preservative, all populations reverted back to the original population profile of resistance (data not shown).

### 3.5 Growth rates of *Z. bailii* in increasing concentrations of preservatives

It has been previously reported that weak-acid preservatives caused a substantial reduction in rate of growth in spoilage yeasts (Stratford and Anslow, 1996; Warth, 1988). In the 96-well microtitre plates, it was necessary to wait until colonies were 0.5–1 mm in size to ensure accurate counting. In the absence of preservatives, this required 2–3 days incubation. At higher concentrations of preservatives, the incubation time required increased up to 12–14 days. It was noted that when the resistant sub-populations were re-inoculated into media containing weak-acids, the slow rate of growth remained unchanged, even though all cells (from that resistant population) then grew. This occurred in sorbic acid, benzoic acid and acetic acid and can be regarded as an indication that preservatives were not being degraded by resistant sub-populations, since this would result in faster growth following removal of preservative.

### 3.6 Cross-resistance in sub-populations of *Z. bailii*

**Fig. 4:**
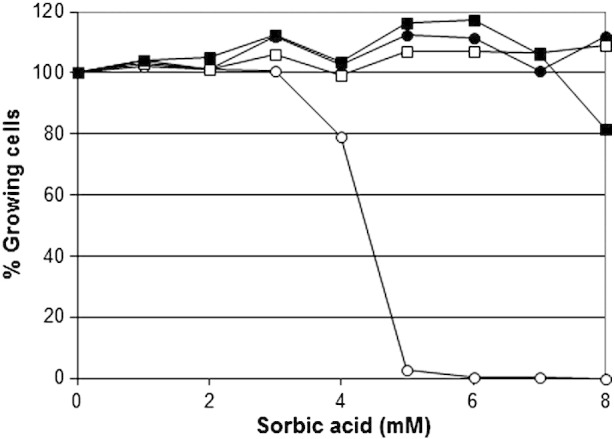
Cross resistance to sorbic acid of individual cells in normal populations of *Z. bailii* NCYC 1766 (open circles) and individual cells in sub-populations grown for 14 days in 350 mM acetic acid (closed squares), 8 mM benzoic acid (open squares) or 6 mM sorbic acid (closed circles) measured in YEPD pH 4.0 over 14 days at 25 °C. Populations of > 8000 cells were grown in 96-well microtitre plates, at 300–600 cells/plate. Similar results were obtained when these sub-populations were re-inoculated into benzoic acid or acetic acid.

Resistant sub-populations were grown over 2 weeks in 6 mM sorbic acid, 8 mM benzoic acid, and 350 mM acetic acid. These populations were then cross-inoculated into all combinations of other preservatives, at a full range of concentrations. Surprisingly, all resistant sub-populations were resistant to all three preservatives tested (Fig. 4). All cell populations grown in 6 mM sorbic acid were fully resistant to sorbic acid, benzoic acid and acetic acid. Similarly, 100% population resistance was obtained in all nine preservative combinations, i.e. cells grown in 8 mM benzoic acid, 6 mM sorbic acid or in 350 mM acetic acid and then inoculated into any weak acid. These data indicate either a common mechanism of action by all three preservatives against *Z. bailii*, or a common resistance mechanism in *Z. bailii* affecting all weak acid preservatives.

### 3.7 ^14^C-Acetic acid uptake in *Z. bailii* and in resistant sub-populations

**Fig. 5:**
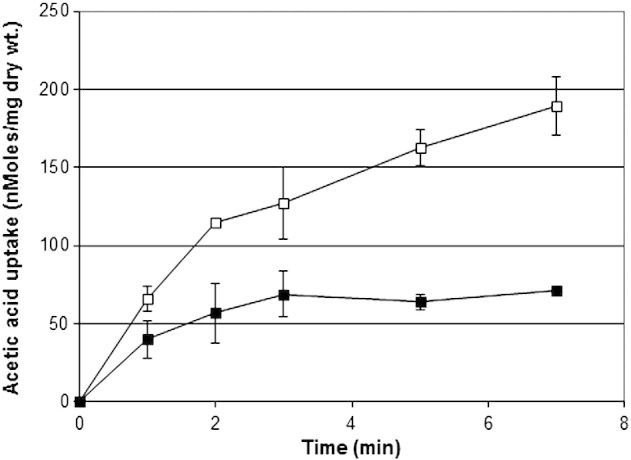
Uptake of ^14^C-acetic acid (30 mM) into cells at the exponential stage of growth in cultures of *Z. bailii* NCYC 1766 in YEPD pH 4.0 (open squares) and sub-populations growing in the presence of 6 mM sorbic acid (closed squares). Data are the means of three independent determinations from separate cultures carried out at 25 °C, with the standard deviations shown for each timed sample.

The data presented have shown that *Z. bailii* is resistant to a variety of weak acids of different structures but not lipophilic alcohols. Furthermore, that resistance is due to heterogeneity within the yeast population, and the resistance to any single acid confers resistance to other (possibly all) weak acids. The simplest hypothesis explaining these data is that there is a mechanism lowering uptake of weak acids in the resistant sub-population, which is non-functional in the bulk population. This would result in a lower cytoplasmic accumulation of all acids and minimise toxic effects, irrespective of any mechanism of action. This hypothesis was tested using uptake of ^14^C-acetic acid, using a low concentration that would not significantly disturb the cytoplasmic pH (Fig. 5). Uptake of acetic acid in populations grown with or without sorbic acid was rapid, reaching a plateau in ~ 3–10 min. This represents the maximum cellular accumulation, a dynamic equilibrium of diffusion into and out from the cell. The initial uptake rate (Fig. 5) reflected the final equilibrium level, but it is the equilibrium level that determines the accumulated concentration of weak-acid. The maximum uptake level in the normal bulk populations of *S. cerevisiae* was marginally higher than the bulk population of *Z. bailii* (data not shown). The resistant *Z. bailii* sub-population cultured in 6 mM sorbic acid showed a considerably reduced uptake of ^14^C-acetic acid, the plateau level of uptake being ~ 4-fold lower than in the bulk *Z. bailii* population. These data confirm that the resistant sub-population of *Z. bailii* took up a lower dose of weak-acid, thus potentially accounting for the high level of resistance.

### 3.8 Intracellular pH in *S. cerevisiae, Z. bailii* and resistant sub-populations

Uptake, and cytoplasmic accumulation, of weak acids in yeast is primarily controlled by the differential between the media pH and intracellular pH. Since the media pH was constant at pH 4.0 in all experiments, it is probable that the lower uptake of acetic acid in the resistant sub-population (Fig. 5) was due to a consistently lower intracellular pH in sub-populations grown in any weak acid. Intracellular pH in cells within the *Z. bailii* population was therefore determined by flow cytometry on CFDASE-treated cells, stained in the growth media to avoid anomalies caused by cell washing.

**Fig. 6:**
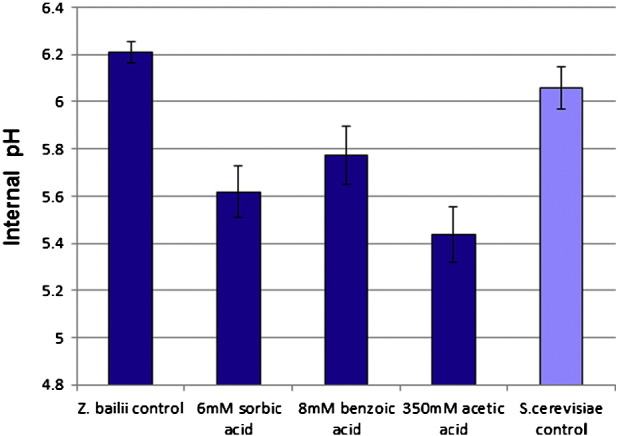
Internal pH measurement of cell populations during exponential growth of *Z. bailii* NCYC 1766 and *S. cerevisiae* BY4741 in YEPD pH 4.0 at 25 °C. Cells were stained with CFDASE in the growth medium at 25 °C and the mean population pHi was determined by fluorescence ratio using flow cytometry. Data are the means of three independent determinations from separate cultures. Control populations were grown without weak acids and *Z. bailii* sub-populations were grown with either 6 mM sorbic acid, 8 mM benzoic acid or 350 mM acetic acid.

Results confirmed that the mean intracellular pH of bulk populations of exponentially-growing *Z. bailii* and *S. cerevisiae* were similar (Fig. 6). In contrast, the mean intracellular pH values of the resistant sub-populations of *Z. bailii* were consistently lower by 0.4–0.8 pH units, depending on the weak acid (sorbic acid p = 0.00271; benzoic acid p = 0.00436; acetic acid p = 0.00857). These data on the lower internal pH of sub-populations grown in weak acid are consistent with the observed reduction in weak-acid uptake (Fig. 5) and are discussed below.

## 4 Discussion

The data presented in this paper confirm the high resistance of all 38 tested strains of *Z. bailii* to weak-acid preservatives. Further tests showed that a representative strain of *Z. bailii* was resistant to a wide variety of lipophilic and hydrophilic weak acids. On average ~ 3-fold more weak-acid was required to inhibit growth of *Z bailii* than *S. cerevisiae*. No enhanced resistance was found to alcohols, aldehydes or esters. Previous reports of *Z. bailii* resistance to alkanols (Fujita et al., 2008; Goswell, 1986; Thomas and Davenport, 1985) remain valid, but comparable resistance is also found in *S. cerevisiae* and therefore those data do not address the issue of relative resistance between *Z. bailii* and other yeasts. Resistance in *Z. bailii* was shown to a wide variety of weak acids. Degradation of acids is unlikely to be a significant factor in resistance, due to the diversity of acid structures (including adamantane carboxylic acid), the lack of growth rate restoration in sub-populations, cross-resistance between dissimilar acids, and earlier studies showing that acid metabolism was insufficient to determine resistance (Warth, 1977). In *Z. bailii*, extreme weak-acid resistance was most probably due to the presence of low numbers of resistant cells in the *Z. bailii* bulk populations. Resistant sub-populations cultured in weak acids showed resistance to extreme concentrations of preservatives, and cross-resistance to other weak-acid preservatives. The diversity of weak-acid structures, and variations in toxicity shown by the MIC values, implies a variety of inhibition mechanisms, and that resistance is due to reduced uptake and accumulation of all weak-acids. Weak acids, unlike alcohols, are accumulated in the cytoplasm at concentrations far higher than the concentrations in the external media. This is due to dissociation of acids into anions in the higher pH of the cytoplasm.

The hypothesis is proposed that extreme resistance in *Z. bailii* is due to the presence of a sub-population of resistant cells and not due to resistance of the bulk population. Resistant cells were shown to have a lower intracellular pH than the weak-acid sensitive bulk population. A lower internal pH, by 0.4–0.8 pH units, would in itself lead to a lower uptake of all weak acids, irrespective of their chemical structures or mechanisms of inhibition. This is supported by an earlier study showing variability in the pH_i_ of individual cells in response to acetic acid (Arneborg et al., 2000). Sensitive cells forming the majority of the *Z. bailii* bulk population, absorbing high concentrations of weak acids, are then likely to die by an apoptosis-like mechanism (Ludovico et al., 2003).

Uptake of weak-acids by yeast at low pH has been shown to be a simple diffusion-based mechanism (Stratford and Rose, 1986; Warth, 1989b). Simple diffusion results in an initial rapid flow into the cell, levelling off as the intracellular concentration equals the external concentration, and a dynamic equilibrium is formed where the inward flow equals the outflow. However, weak acids also form a pH-dependent equilibrium between undissociated acid molecules and dissociated anions, e.g. acetic acid and acetate. At low pH, molecular acids predominate whereas at neutral pH anions are in the great majority. The pH at which the ratio is 50/50 is termed the pK_a_ and the ratio proportions can be calculated using the Henderson–Hasselbalch equation, where [A^−^] and [HA] are the anion and acid concentrations, respectively.

$$\mathrm{pH}={\mathrm{pK}}_{a}+\log\left[A^{-}\right]/\left[\mathrm{HA}\right]$$

**Fig. 7:**
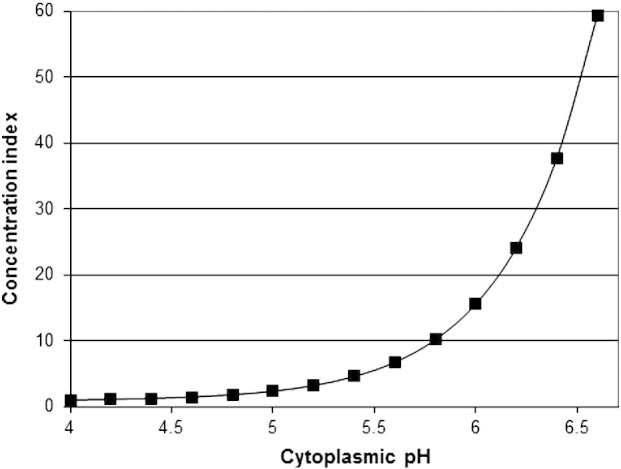
Increase in accumulation (concentration index) of sorbic acid (pK_a_ 4.76) in cells with an internal pH rising from pH 4.0 to pH 6.6, in an external medium of pH 4.0, calculated using the Henderson/Hasselbach equation, assuming infinite buffering capacity and no pH alteration caused by accumulation.

For both sorbic and acetic acids the pK_a_ is 4.76, giving the ratio at pH 4.0 to be 85.3% acid and, at pH 6.6, to be 1.4% acid. Assuming infinite buffering capacity and no pH alteration caused by accumulation,1 mM extracellular weak acid at pH 4.0 (0.85 mM acid) will therefore diffuse into the cell until the intracellular acid concentration is also 0.85 mM, in equilibrium with an anion concentration of ~ 60 mM, giving a 60-fold concentration within the cell (intracellular pH 6.6). Fig. 7 shows the calculated concentration index for sorbic/acetic acids at different intracellular pH. While at pH_i_ 6.2, these acids are concentrated by 24-fold, at an intracellular pH of 5.6, these acids are concentrated by only 6.7-fold. This would be predicted to result in a 3.6-fold lower accumulation of preservative in the resistant cells. This would be sufficient to account for the observed extreme resistance of the *Z. bailii* sub-population and the sensitive bulk population. This ratio is also remarkably close to the observed ratio of weak-acid resistance concentrations (2.98—Table 2) between the *Z. bailii* population (long resistance tail) and *S. cerevisiae* population (short resistance tail). A 3-fold increase in concentration of weak acid applied to *Z. bailii*, would be prediced to result in a similar internal concentration to that resulting from a 1-fold concentration applied to *S. cerevisiae*.

Several previous studies have considered the significance of rate of uptake of weak acids into *Z. bailii* as a cause of resistance (Warth, 1977, 1989b). This is not likely to be a factor affecting resistance. Initial rate of diffusion may be related to amount of uptake, but it is probable that the absorbed dose of a toxin that determines toxicity not the rate of uptake. Earlier studies have also considered the behaviour of “adapted” cells of *Z. bailii* (Warth, 1989b). These cells are almost certainly not adapted but resistant sub-populations of *Z. bailii* cells grown under selection pressure of the weak acids. The pH_i_ of *Z. bailii* cells growing in preservatives was previously noted as reduced in sorbic acid (Cole and Keenan, 1987) and acetic acid (Dang et al., 2012), but the significance of this was not realised at the time.

Until now, reduction in pH_i_ was assumed to be caused by weak-acid acidification, rather than as a resistance mechanism. Certainly, lowering of pH_i_ will have deleterious effects on cellular metabolism, particularly to values below the pH optimum for many enzymes (Pearce et al., 2001). It is possible that a compromise may be beneficial; a moderate lowering of pH_i_ will still enable sufficient enzyme activity for growth, while preventing the accumulation of toxic levels of weak acids. It has been observed for many years that one universal effect of weak acids at sub-inhibitory levels, was to cause a slow growth rate and low cell yield (Stratford and Anslow, 1996). Until now, this has been assumed to be caused by the weak acids, but it is also possible that this is caused by a resistance mechanism. The relatively low pH_i_ in the sub-population would, in that scenario, minimise weak-acid uptake but would also reduce growth rate due to inhibition of metabolism. We showed that the properties of the weak-acid resistant sub-population of *Z. bailii* are not stably inherited, indicating that existence of the sub-population is an example of phenotypic heterogeneity within a population (Avery, 2006). Several factors are known to contribute to phenotypic heterogeneity, which is acknowledged to have an important impact on bioprocesses (Avery, 2006; Fernandes et al., 2011), but we cannot comment further on the contributory mechanisms here.

Careful consideration of the facts suggests that a lowering of pH_i_ cannot alone form a resistance mechanism. Lower pH_i_ can be caused directly by addition of high concentrations of acetic acid, but this does not cause 100% of cells to become resistant. This suggests that resistant cells have a lowered pH_i_ before weak acid addition and that they also have an adjusted metabolism to allow growth, albeit slower, in an acidified cytoplasm. Further research will be required to uncover the scope and mechanism of such changes.

The following are the supplementary data related to this article.

Supplementary Data Table 1 Comparison of resistance of *Zygosaccharomyces bailii* NCYC 1766 and *Saccharomyces cerevisiae* strain BY4741 to 87 chemical inhibitors. Inhibitors are grouped by chemical structure and listed with their molecular weight (M.W.) and partition coefficient (cLogP_oct_). MIC values (mM) were determined in YEPD pH 4.0 at 10^3^ cells/ml over 14 days at 25 °C and are presented with the MIC ratio of *Z. bailii/S.cerevisiae*. Equal resistance is indicated by 1, enhanced *Z. bailii* resistance is indicated by higher values.

## Figures and Tables

**Table 1:** Strains of *Zygosaccharomyces bailii* and *Saccharomyces cerevisiae* used in this study and their origins. NCYC strains are available from the National Collection of Yeast Cultures, Norwich UK. Others were collected (strain numbers) over several years from the food industry. All strains were confirmed in identity by D1/D2 rDNA sequencing. Weak-acid preservative resistance, sorbic acid, benzoic acid and acetic acid, was measured in YEPD pH 4.0 at 10^3^ cells/ml and incubated at 25 °C for 2 weeks at pH 4.0. Numbers provided in the columns headed by sorbic, benzoic and acetic are the lowest concentration of weak acids (mM) to completely inhibit growth.

Strain	Species	Origin	Sorbic	Benzoic	Acetic
BY4741	*S. cerevisiae*	Euroscarf	3	2.9	120
NCYC 3253	*S. cerevisiae*	Spoilage, soft drink UK	3.5	3.4	145
4	*Z. bailii*	Spoilage, canned ice tea USA	6.54	8.5	550
5	*Z. bailii*	Spoilage, canned fruit USA	6.55	8	533
6	*Z. bailii*	Spoilage, bottled ice tea USA	7.46	9.12	545
7	*Z. bailii*	Spoilage, preserved fruit punch USA	6.67	8.13	475
8	*Z. bailii*	Spoilage, soft drink USA	6.68	8.5	467
9	*Z. bailii*	Spoilage, carbonated orange drink USA	8.04	8.13	468
10	*Z. bailii*	Spoilage, soft drink USA	6.35	8.33	483
11	*Z. bailii*	Spoilage, soft drink USA	7	9.13	466
12	*Z. bailii*	Spoilage, carbonated orange drink USA	8.09	9.75	468
13	*Z. bailii*	Spoilage, soft drink USA	7.06	10.12	467
15	*Z. bailii*	Spoilage, salad dressing Netherlands	7.44	8.88	444
16	*Z. bailii*	Spoilage, salad dressing Netherlands	7.13	7.75	400
17	*Z. bailii*	Spoilage, salad dressing UK	6.69	8.87	517
21	*Z. bailii*	Spoilage, herring in tomato sauce UK	4.55	7.65	275
52	*Z. bailii*	Spoilage, salad dressing Netherlands	5.83	9.13	567
80	*Z. bailii*	Spoilage, Mexican Topping sauce UK	6.2	9.75	450
105	*Z. bailii*	Spoilage, tomato sauce UK	7.97	8.37	475
106	*Z. bailii*	Spoilage, tomato sauce UK	7.75	8.11	470
107	*Z. bailii*	Spoilage, tomato sauce UK	7.34	8.2	400
108	*Z. bailii*	Spoilage, tomato sauce UK	7.83	8.14	466
112	*Z. bailii*	Spoilage, ice tea Belgium	6.6	8.25	450
114	*Z. bailii*	Spoilage, ice tea Belgium	6.3	9.25	450
119	*Z. bailii*	Spoilage, soft drink Netherlands	8.75	9.75	500
280	*Z. bailii*	Spoilage, soft drink South Africa	8.4	9	400
362	*Z. bailii*	Factory isolate Turkey	6.8	8.3	440
475	*Z. bailii*	Factory isolate Brazil	7	8	450
503	*Z. bailii*	Kombucha, fermented tea UK	8.5	9.5	530
505	*Z. bailii*	Kombucha, fermented tea UK	8.8	10	450
593	*Z. bailii*	Factory isolate Philippines	4.5	8.25	450
595	*Z. bailii*	Spoilage, dried fruit Spain	7.9	10.1	500
DBVPG 6924	*Z. bailii*	Anne Vaughn-Martini, USA	8.5	9.5	580
NCYC 1766	*Z. bailii*	Spoilage, Blackcurrant & Grape UK	7.62	8.65	467
NCYC 563	*Z. bailii*	Spoilage, sorghum brandy	5.75	7.75	375
NCYC 3378	*Z. bailii*	Factory isolate Philippines	7.65	9.15	550
NCYC 3407	*Z. bailii*	Spoilage, lemon tea UK	6.19	9.12	484
NCYC 3410	*Z. bailii*	Spoilage, herring in tomato sauce UK	6.13	8.12	383
NCYC 3414	*Z. bailii*	Spoilage, orange concentrate UK	5.85	6.25	450
NCYC 3590	*Z. bailii*	Spoilage, jam Sweden	9.45	11	390
		*Z. bailii* Mean	7.10	8.75	465.39
		*Z. bailii* S.D.	1.11	0.89	59.57

**Table 2:** Summary of data comparing resistance of *Zygosaccharomyces bailii* NCYC 1766 and *Saccharomyces cerevisiae* strain BY4741 to 87 chemical inhibitors. Full data are presented in Supplementary Data Table 1. Chelating weak acids were EDTA, citric, succinic, and lactic acids. MIC values (mM) were determined in YEPD pH 4.0 at 10^3^ cells/ml over 14 days at 25 °C. The ratio of MICs of *Z. bailii/S.cerevisiae* was determined for each compound and averaged (Mean Ratio). Equal resistance is indicated by 1, enhanced *Z. bailii* resistance is indicated by higher values.

Inhibitor	Number tested	Mean MIC ratio	S.D.
Chelators	4	1.06	0.27
Weak acids	30	2.98	1.27
Aldehydes	16	1.1	0.34
Ketones	4	0.99	0.12
Alcohols	18	1.13	0.39
Ethers	4	1.12	0.24
Esters	11	0.92	0.25
