# Supplementary material for: Extreme resistance to weak-acid preservatives in the spoilage yeast Zygosaccharomyces bailii
Source: Int J Food Microbiol. 2013 Aug 16;166(1):126–34. doi: 10.1016/j.ijfoodmicro.2013.06.025 (PMC3759830; doi:10.1016/j.ijfoodmicro.2013.06.025)
Supplement: Supplementary Data Table 1 — Comparison of resistance of Zygosaccharomyces bailii NCYC 1766 and Saccharomyces cerevisiae strain BY4741 to 87 chemical inhibitors. Inhibitors are grouped by chemical structure and listed with their molecular weight (M.W.) and partition coefficient (cLogPoct). MIC values (mM) were determined in YEPD pH 4.0 at 103 cells/ml over 14 days at 25 °C and are presented with the MIC ratio of Z. bailii/S.cerevisiae. Equal resistance is indicated by 1, enhanced Z. bailii resistance is indicated by higher values. [file mmc1.doc]

**Supplementary Data Table 1.**

Comparison of resistance of *Zygosaccharomyces bailii* NCYC 1766 and *Saccharomyces cerevisiae* strain BY4741 to 87 chemical inhibitors. Inhibitors are grouped by chemical structure and listed with their molecular weight (M.W.) and partition coefficient (cLogP_oct_). MIC values (mM) were determined in YEPD pH 4.0 at 10^3^ cells/ml over 14 days at 25°C and are presented with the MIC ratio of *Z. bailii/S.cerevisiae*. Equal resistance is indicated by 1, enhanced *Z. bailii* resistance is indicated by higher values.

|  | **Inhibitor** | **M.W.** | **cLogP_oct_** | ***Z.bailii*** | ***S.cerevisiae*** | **Ratio** |
| --- | --- | --- | --- | --- | --- | --- |
| **Acids** | Acetic acid | 60.05 | -0.19 | 425 | 130 | 3.27 |
|  | Acrylic acid | 72.06 | 0.35 | 52 | 23 | 2.26 |
|  | Adamantanecarboxylic acid | 180.25 | 2.46 | >2.4 | 1 | 2.40 |
|  | Adamantaneacetic acid | 194.27 | 4.12 | >1.8 | 0.7 | 2.57 |
|  | Benzoic acid | 122.12 | 1.88 | 11.5 | 3 | 3.83 |
|  | Butyric acid | 88.11 | 0.86 | 70 | 26 | 2.69 |
|  | Cinnamic acid | 148.16 | 2.24 | 1.3 | 0.7 | 1.86 |
|  | Citric acid | 192.13 | -2.00 | 1125 | 1125 | 1.00 |
|  | Crotonic acid | 86.09 | 0.88 | 51 | 23 | 2.22 |
|  | Cyclohexanecarboxylic acid | 128.17 | 1.84 | 12 | 4 | 3.00 |
|  | Cyclohexanepropionic acid | 158.24 | 0.16 | 2.1 | 0.63 | 3.33 |
|  | Cyclopropanecarboxylic acid | 86.09 | 0.16 | 142 | 51 | 2.78 |
|  | 2,4-Dinitrophenol | 184.11 | 1.40 | 0.42 | 0.15 | 2.80 |
|  | EDTA | 292.25 | -1.93 | 30 | 35 | 0.86 |
|  | Formic acid | 46.03 | -0.54 | 108 | 38 | 2.84 |
|  | Heptanoic acid | 130.19 | 2.45 | 2.5 | 1.15 | 2.17 |
|  | Hexanoic acid | 116.16 | 1.92 | 6.4 | 2.6 | 2.46 |
|  | Hydrocinnamic acid | 150.18 | 1.90 | 9.5 | 2.8 | 3.39 |
|  | p-Hydroxybenzoic acid | 138.12 | 0.83 | 685 | 151 | 4.54 |
|  | Ferulic acid | 194.19 | 1.42 | 140 | 30 | 8.75 |
|  | Lactic acid | 90.08 | -0.73 | 2100 | 1450 | 1.45 |
|  | Methacrylic acid | 86.09 | 0.66 | 38 | 12.5 | 3.04 |
|  | Nonanoic acid | 158.24 | 3.51 | 0.85 | 0.35 | 2.43 |
|  | Octanoic acid | 144.21 | 2.98 | 1.15 | 0.51 | 2.25 |
|  | 3-Phenylpropiolic acid | 146.15 | 1.78 | 13 | 6 | 2.17 |
|  | 2-Phenylpropionic acid | 150.18 | 1.72 | 33 | 10 | 3.30 |
|  | Propionic acid | 74.08 | 0.33 | 170 | 60 | 2.83 |
|  | Salicylic acid | 138.12 | 2.19 | 7.7 | 3.7 | 2.08 |
|  | Sorbic acid | 112.13 | 1.51 | 7 | 2.9 | 2.41 |
|  | trans-Styrylacetic acid | 162.19 | 2.18 | 3 | 0.7 | 4.29 |
|  | Succinic acid | 118.09 | -0.53 | 1200 | 1300 | 0.92 |
|  | Tiglic acid | 100.12 | 1.19 | 20 | 10 | 2.00 |
|  | Valeric acid | 102.13 | 1.39 | 20 | 9 | 2.22 |
|  | Vinylacetic acid | 86.09 | 0.58 | 77 | 25 | 3.08 |
| **Aldehydes** | Acetaldehyde | 44.05 | -0.22 | 23 | 23 | 1.00 |
|  | o-Anisaldehyde | 136.15 | 1.78 | 5 | 5 | 1.00 |
|  | p-Anisaldehyde | 136.15 | 1.78 | 7.7 | 11.5 | 0.67 |
|  | Benzaldehyde | 106.12 | 1.50 | 7.6 | 11.4 | 0.67 |
|  | Butanal | 72.11 | 0.83 | 20 | 20 | 1.00 |
|  | Cinnamaldehyde | 132.16 | 2.05 | 0.51 | 0.34 | 1.50 |
|  | 2,4-Decadienal | 152.24 | 3.27 | 0.152 | 0.084 | 1.81 |
|  | 4-Ethoxybenzaldehyde | 150.18 | 2.31 | 5.1 | 4 | 1.28 |
|  | Formaldehyde | 30.03 | -0.69 | 2.6 | 2.2 | 1.18 |
|  | Heptanal | 114.19 | 2.42 | 1.12 | 1.25 | 0.90 |
|  | Hexanal | 100.16 | 1.89 | 2 | 2.3 | 0.87 |
|  | Cuminaldehyde | 148.20 | 2.92 | 1.7 | 1 | 1.70 |
|  | α-Methylcinnamaldehyde | 146.19 | 2.36 | 1.14 | 1.45 | 0.79 |
|  | Pentanal | 86.13 | 1.36 | 4.4 | 4.2 | 1.05 |
|  | Propanal | 58.08 | 0.30 | 15 | 15 | 1.00 |
|  | Salicylaldehyde | 122.12 | 1.81 | 0.76 | 0.63 | 1.21 |
| **Ketones** | Acetone | 58.08 | -0.21 | 2.23 | 2.68 | 0.83 |
|  | 2-Acetylfuran | 110.11 | 0.76 | 80 | 77 | 1.04 |
|  | Benzophenone | 182.22 | 3.18 | 0.95 | 0.86 | 1.10 |
|  | Diacetyl | 86.09 | -1.37 | 1.71 | 1.71 | 1.00 |
| **Alcohols** | Butanol | 74.12 | 0.82 | 215 | 260 | 0.83 |
|  | Cinnamyl alcohol | 134.18 | 1.61 | 7.6 | 8.5 | 0.89 |
|  | Citronellol | 156.27 | 3.25 | 0.76 | 0.51 | 1.49 |
|  | m-Cresol | 108.14 | 1.97 | 7.7 | 11.5 | 0.67 |
|  | Cumic alcohol | 150.22 | 2.53 | 4.4 | 4.4 | 1.00 |
|  | Decanol | 158.28 | 4.00 | 0.23 | 0.23 | 1.00 |
|  | 3,7-Dimethyloctanol | 158.28 | 3.74 | 2.4 | 0.96 | 2.50 |
|  | Ethanol | 46.07 | -0.24 | 2100 | 1900 | 1.11 |
|  | Eugenol | 164.20 | 2.40 | 3.4 | 3.4 | 1.00 |
|  | Fenchyl alcohol | 154.25 | 3.10 | 3.4 | 2.6 | 1.31 |
|  | Heptanol | 116.20 | 2.41 | 5.1 | 4.2 | 1.21 |
|  | Hexanol | 102.18 | 1.88 | 17 | 17 | 1.00 |
|  | Methanol | 32.04 | -0.76 | 3800 | 3800 | 1.00 |
|  | Nonanol | 144.26 | 3.47 | 0.6 | 0.57 | 1.05 |
|  | Octanol | 130.23 | 2.94 | 2.3 | 1.9 | 1.21 |
|  | cis-5-Octenol | 128.21 | 2.45 | 3 | 3 | 1.00 |
|  | Pentanol | 88.15 | 1.35 | 75 | 60 | 1.25 |
|  | Propanol | 60.10 | 0.29 | 530 | 610 | 0.87 |
| **Ethers** | p-Allylanisole | 148.20 | 3.13 | 2.3 | 2.3 | 1.00 |
|  | Benzyl-ether | 198.26 | 3.30 | 100 | 100 | 1.00 |
|  | Eugenylmethyl ether | 178.23 | 2.87 | 3.4 | 2.3 | 1.48 |
|  | Phenyl ether | 170.21 | 4.24 | 26 | 26 | 1.00 |
| **Esters** | Ethyl propionate | 102.13 | 1.24 | 63 | 114 | 0.55 |
|  | Ethyl pyruvate | 116.12 | 0.16 | 12.8 | 12.8 | 1.00 |
|  | Isoamyl acetoacetate | 172.22 | 1.79 | 7.7 | 5.1 | 1.51 |
|  | Methyl acetate | 74.08 | 0.18 | 510 | 572 | 0.89 |
|  | Methyl butyrate | 102.13 | 1.24 | 60 | 80 | 0.75 |
|  | Methyl heptanoate | 144.21 | 2.83 | 4.2 | 5 | 0.84 |
|  | Methyl hexanoate | 130.19 | 2.30 | 10 | 10 | 1.00 |
|  | Methyl nonanoate | 172.27 | 3.88 | 40 | 38 | 1.05 |
|  | Methyl propionate | 88.11 | 0.71 | 256 | 250 | 1.02 |
|  | Methyl valerate | 116.16 | 1.77 | 20 | 25 | 0.80 |
|  | Propyl butyrate | 130.19 | 2.30 | 10 | 15 | 0.75 |
